# Immune Modulation Plus Tumor Ablation: Adjuvants and Antibodies to Prime and Boost Anti-Tumor Immunity *In Situ*

**DOI:** 10.3389/fimmu.2021.617365

**Published:** 2021-04-14

**Authors:** Renske J. E. van den Bijgaart, Fabian Schuurmans, Jurgen J. Fütterer, Marcel Verheij, Lenneke A. M. Cornelissen, Gosse J. Adema

**Affiliations:** ^1^ Radiotherapy & OncoImmunology Laboratory, Department of Radiation Oncology, Radboud University Medical Center, Nijmegen, Netherlands; ^2^ Department of Medical Imaging, Radboud University Medical Center, Nijmegen, Netherlands; ^3^ Department of Robotics and Mechatronics, University of Twente, Enschede, Netherlands

**Keywords:** tumor ablation, immune activation, *in situ* cancer vaccination, multifunctional antibodies, combination therapy

## Abstract

*In situ* tumor ablation techniques, like radiotherapy, cryo- and heat-based thermal ablation are successfully applied in oncology for local destruction of tumor masses. Although diverse in technology and mechanism of inducing cell death, ablative techniques share one key feature: they generate tumor debris which remains *in situ*. This tumor debris functions as an unbiased source of tumor antigens available to the immune system and has led to the concept of *in situ* cancer vaccination. Most studies, however, report generally modest tumor-directed immune responses following local tumor ablation as stand-alone treatment. Tumors have evolved mechanisms to create an immunosuppressive tumor microenvironment (TME), parts of which may admix with the antigen depot. Provision of immune stimuli, as well as approaches that counteract the immunosuppressive TME, have shown to be key to boost ablation-induced anti-tumor immunity. Recent advances in protein engineering have yielded novel multifunctional antibody formats. These multifunctional antibodies can provide a combination of distinct effector functions or allow for delivery of immunomodulators specifically to the relevant locations, thereby mitigating potential toxic side effects. This review provides an update on immune activation strategies that have been tested to act in concert with tumor debris to achieve *in situ* cancer vaccination. We further provide a rationale for multifunctional antibody formats to be applied together with *in situ* ablation to boost anti-tumor immunity for local and systemic tumor control.

## Introduction

Vaccines have been extremely successful in preventing infectious diseases by training the immune system to recognize and destroy pathogens. Conventional vaccines comprise of antigen(s) often supplemented with immune adjuvants to support the induction of an effective immune response. Besides, adjuvants can function as a slow release system, ensuring prolonged and continuous presentation and stimulation of the immune system (1). The application of vaccines to cancer is an obvious extension of their utility, and many diverse vaccination strategies are under development. An interesting novel approach is the *in vivo* loading and activation of dendritic cells (DCs) with tumor antigens released following *in situ* tumor ablation.

**Figure 1:**
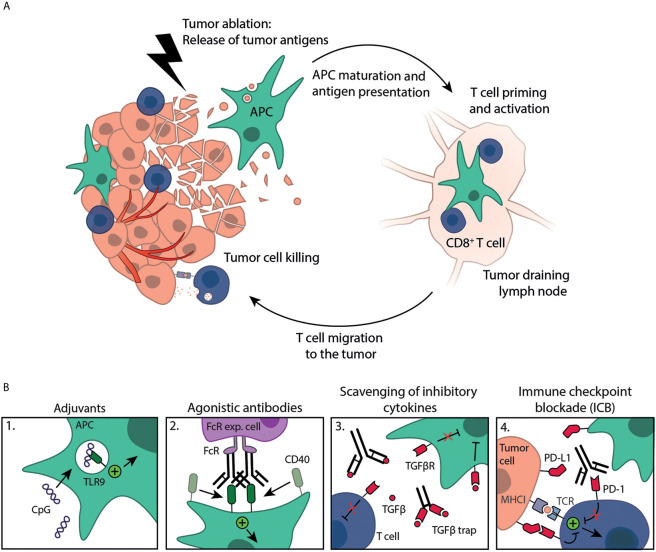
Immune activation strategies plus tumor ablation to create *in situ* cancer vaccines. **(A)** Tumor ablation results in the release of tumor antigens available for uptake by antigen-presenting cells (APC), such as DCs. These antigens are (cross-)presented on MHC molecules to T cells in the dLN, resulting in T cell priming and activation. Activated T cells subsequently migrate to the destructed tumor, as well as distant metastases, where they kill remaining tumor cells. **(B)** Immune response induction is boosted by exogenous administration of immune stimulating compounds like 1. adjuvants (e.g. CpG) or 2. agonistic antibodies (e.g. anti-CD40 mAb, crosslinking by Fc-receptor expressing cells) that can work synergistically with tumor ablation in creating effective, mature DCs. Furthermore, several approaches can be exploited to counteract the immunosuppressive TME, such as 3. scavenging of inhibitory cytokines (e.g. anti-TGFβ mAb or TGFβ trap) or 4. immune checkpoint blockade (ICB, e.g. anti-PD-1 mAb), both to enhance the anti-tumor immune response.

Tumor ablation techniques are successfully applied for the treatment of different malignancies. Although diverse in technology and mechanism of action, all ablative techniques lead to *in situ* availability of ablated tumor material (**Figure 1A**). The tumor debris released upon ablation functions as an antigen depot representing the tumors’ antigenic repertoire. Together with the simultaneous release of bioactive molecules, such as damage-associated molecular patterns (DAMPs), this has led to the concept of *in situ* cancer vaccination. Indeed, tumor antigens were observed in DCs residing in draining lymph nodes (dLNs) following ablation (2). Immune responses induced by ablation as stand-alone treatment are documented, however, tend to be incapable of evoking robust sustainable anti-tumor immunity. This is further evidenced by the scarce reports of spontaneous regression of untreated distant metastatic sites following ablation, the so-called ‘abscopal effect’ (3, 4). Therefore, it has been proposed by us and others to initiate and boost ablation-induced anti-tumor immunity by combining ablation with immune activation strategies (5–7). An outstanding question in the field remains which immune activation strategies effectively combine with *in situ* tumor ablation.

Examples of immune activation strategies that are mostly applied together with tumor ablation include stimulation with pattern recognition receptor (PRR) agonists, adjuvants and agonistic antibodies. However, tumors have evolved mechanisms to create an immunosuppressive tumor microenvironment (TME), parts of which may admix with the antigen depot upon ablation. Development of a successful *in situ* cancer vaccine thus requires immune activation strategies to boost immunity and approaches that counteract the immunosuppressive TME like monoclonal antibodies (mAbs) against inhibitory immune checkpoints, inhibition of immunosuppressive cells like regulatory T cells (Treg) or myeloid derived suppressor cells (MDSC) or by scavenging anti-inflammatory cytokines, such as transforming growth factor beta (TGFβ).

Delivery of these immunomodulators to the relevant locations, i.e. the tumor and tumor dLNs, is often essential for enhancing anti-tumor specific immune responses following ablation. Targeting also mitigates potential toxic side-effects. Antibodies can exhibit tumor targeting abilities, either through their specificity for tumor antigens or ablation-associated factors, such as extracellular DNA and histones. Recent advances in antibody engineering enabled the creation of novel antibody formats with multiple functions, such as bispecific antibodies and protein-linked antibodies (8). Multifunctional antibodies create new opportunities to enhance anti-tumor immunity following *in situ* tumor ablation techniques.

Here, we review immune activation strategies and approaches that counteract the immunosuppressive TME that have been combined with *in situ* tumor ablation. Furthermore, we postulate new combination strategies involving multifunctional antibody formats to be applied together with *in situ* ablation to boost the anti-tumor immunity for local and systemic tumor control.

## *In Situ* Tumor Ablation

During the last decades, there has been widespread interest in the development and refinement of ablation techniques for local treatment of cancers. The primary goal of tumor ablation is to destroy malignant cells within a designated volume through the local deposition of energy *via* different approaches, e.g. ionizing radiation or extreme temperatures. Radiotherapy (RT) has been a strong pillar in cancer therapy and the majority of cancer patients undergo RT at one point during treatment (9). The anti-tumor efficacy of RT has been attributed to its capacity to induce DNA damage, as well as through increased recognition of tumor cells by the immune system. Stereotactic ablative body radiotherapy (SABRT) allows for the delivery of ablative radiation doses. An exciting development in radiation oncology is the magnetic resonance linear accelerator (MR-Linac) which enables high precision ablative RT under real-time MR-guidance, providing better target control while sparing the surrounding healthy tissues including LNs (10). MR-guided RT is a promising tool to answer key questions in the field of immuno-radiobiology, and will help to understand how to bring dose and fractionation schedules into an immunologically active range.

Different from RT, most other tumor ablation techniques rely on extreme temperatures for cellular destruction. Cryoablation applies extremely cold temperatures, whereas heat-based thermal ablation modalities, including radiofrequency ablation (RFA), microwave ablation (MWA), laser ablation and thermal high-intensity focused ultrasound (HIFU) employ different sources of energy to heat the target region (7, 11). Cells in the core of the ablation zone are subjected to lethal temperatures; up to -180°C for cryoablation inducing hypothermic necrosis and >60°C for thermal ablation resulting in protein denaturation and coagulative necrosis. Cells in the periphery of the ablation zones are exposed to sublethal temperatures and either undergo apoptotic cell death or are able to recover from reversible injury (12). In contrast to thermal HIFU ablation, HIFU can be used to generate mechanical damage as a result of acoustic cavitation, with minimal thermal damage, also known as (boiling) histotripsy or mechanical HIFU (12–14).

Besides their ability to kill tumor cells, ablation modalities unveil an array of tumor antigens. Several studies have emphasized the importance of neoantigens arising due to tumor-specific DNA mutations in the recognition of tumor cells by the immune system (15, 16). Each ablation technique results in a unique tumor antigenic fingerprint. Heat-based thermal ablation results in protein denaturation and coagulative necrosis, possibly reducing the availability of intact tumor antigens for the immune system. Furthermore, the coagulative necrosis destroys the structure and vasculature of tumors, thereby affecting the ability of immune cells to reach and interact with the antigen depot (17). Mechanical HIFU, on the other hand, will result in complete liquification of the tissue, which is effectively removed *via* drainage or absorbed as part of the physiologic healing response (18, 19). For cryoablation it has been reported that many native antigen structures are preserved (20). Furthermore, cryoablation has shown to induce polyclonality and intra-tumoral T cell repertoire remodeling (21). How each ablation technique affects handling and processing of antigenic materials by antigen-presenting cells (APCs) and which ablation technique results in the most effective release of immunogenic (neo)antigens remains to be investigated.

During efforts of the body to clear this tumor debris, there is a time frame in which the immune system can be triggered towards antigens from the antigen depot (**Figure 1A**). In fact, the presence of the antigen depot is a prerequisite for the development of an anti-tumor immune response, as protective immunity failed to develop upon surgical removal of cryoablated tumors (2). Cytokines and endogenous danger signals, such as DAMPs, are readily released from the ablated tumor, which may contribute to immune activation. On the other hand, ablation will also lead to a physiological wound healing response that regulates and maintains immunological tolerance toward the damaged tissue. In practice, ablation induced immunomodulation alone appears (often) insufficient to generate consistent protective anti-tumor immunity. Therefore, interest has shifted towards exploring the potential synergy between ablative techniques and immune activation strategies. Strong systemic immunity will be critical for controlling residual disease at the site of ablation and for eradicating distant metastases.

## Antigen Presenting Cells and Immune Activating Strategies

DCs, the most potent APCs of the immune system, have the unique ability to initiate and direct immune responses. DCs in the vicinity of or recruited to the local ablation site acquire and process tumor antigens, and subsequently present them to naïve T cells (22). Alternatively, tumor antigens may passively enter the circulation or lymphatics and can be transported to LNs where they can be taken up by LN-resident DCs. DCs can cross-present peptides derived from such extracellular antigens to MHC I restricted CD8^+^ T cells. In addition to the initial interaction between the TCR and MHC-molecules on DCs, co-stimulation (signal 2) and cytokines (signal 3) are important for initiation of antigen-specific T cells. Thus, proper DC maturation is essential for efficient immune response induction. The ability to load and mature DCs directly *in situ* by tumor ablation plus immune activation is thus an appealing strategy to develop a cancer vaccine.

### Adjuvants

Adjuvants can boost the magnitude and duration of the adaptive immune response. One of the ways through which adjuvants act is by serving as, or inducing, DAMPs and/or pathogen-associated molecular patterns (PAMPs) that trigger PRRs on immune cells resulting in their activation. Alternatively, adjuvants can function as slow release system. Although numerous different adjuvants exist, we will focus on nucleic acid-sensing PRR agonists, as well as the potential of saponin-based adjuvants (SBAs) applied in combination with *in situ* tumor ablation.

#### Toll-Like Receptor (TLR) Agonists

TLR triggering is one of the most potent inducers of DC maturation *in vivo* as evidenced by their capacity to upregulate co-stimulatory molecules and enhanced production of pro-inflammatory cytokines needed for DC-mediated T cell priming. The nucleic acid-sensing TLRs include TLR9, TLR3 and TLR7/8. CpG oligodeoxynucleotides (CpG) are short unmethylated single stranded-synthetic DNA molecules, which were one of the first adjuvants to be combined with *in situ* tumor ablation (2). Pioneering work combining CpG with cryoablation in a B16OVA melanoma model showed the induction of long-term immune memory, evidenced by a 50% survival of mice subjected to a re-challenge. The survival benefit was absent in the single treatment groups. Additional studies showed the anti-tumor effect is dependent on plasmacytoid DCs (pDCs), which stimulate the ability of conventional type 1 DCs (cDC1s) to prime naïve CD8^+^ T cells (23, 24). A prerequisite for the synergy between cryoablation and CpG is the co-localization of the antigen and CpG within a DC. Therefore, the timing and location of CpG administration relative to the release of tumor antigens by tumor ablation is of importance for protective anti-tumor immunity (25, 26). The beneficial effects of CpG with cryoablation have also been observed in a mammary adenocarcinoma model (27). CpG combinations with other ablative therapies, such as RT and HIFU, have also proven successful (28–30). Interestingly, the combination of thermal HIFU ablation of mammary adenocarcinoma tumors with CpG plus anti-PD-1 increased the number of unique CDR3 rearrangements in the T cell repertoire at distal tumors, indicating the generation of T cells specific for a broad range of different tumor antigens (30).

The synthetic dsRNA analog polyinosinic-polycytidylic acid (Poly-IC) is a ligand for TLR3 and has shown promising preclinical results in combination with a minimal 9 Gy single dose RT. The combination treatment greatly reduced tumor growth at primary and abscopal sites and enhanced survival in different mouse models (31–33). The Poly-IC plus RT combination treatment of A20 lymphoma tumors plus intra-tumoral (i.t.) FLT3L injections further increased DC recruitment and synergistically induced adaptive anti-tumor immunity (33). Mechanistic studies revealed that RT increased serum levels of high mobility group box 1 (HMGB1), a known DAMP *in vivo*. HMGB1 potentiated the Poly-IC induced DC maturation, demonstrating the potential of DAMP plus TLR adjuvant combination strategies (33).

TLR7/8 agonists gained fame through Imiquimod, an adjuvant formulation topically applied in the treatment of skin cancers (34). Topical Imiquimod application, as well as systemic administration (encapsulated in nanoparticles (NP)), in combination with cryoablation or RT, resulted in improved tumor control at primary and distant sites in numerous murine cancer models (35–38). As with CpG, TLR7 agonists are believed to act primarily through activation of pDCs (39). The results of a phase I/II clinical trial investigating the efficacy of topical imiquimod application to breast cancers skin metastases in conjunction with RT are currently on their way (NCT01421017). Altogether, TLR agonists can be employed as powerful adjuvants along with ablation to generate effective anti-tumor immunity.

#### STING Agonists

DNA normally resides in the nucleus and mitochondria; hence, its presence in the cytoplasm serves as a danger signal. This aberrant localization of DNA is sensed by the DNA binding enzyme cyclic GMP-AMP synthase (cGAS). Upon recognition, cGAS dimerizes and stimulates the production of cyclic-GMP-AMP (cGAMP) which can directly bind stimulator of interferon genes (STING) resulting in type I interferon (IFN) production (40). Cytoplasmic DNA sensing through the cGAS-STING pathway plays a pivotal role in APC activation following phagocytosis (41).

Synthetic analogues of 2’3’-cGAMP, a stable variant of the second messenger produced by cGAS are used as STING-activating agents. Combinations of such analogues with ablative therapy are scarce and limited to one study by Deng et al. which showed i.t. injection of 2’3’-cGAMP in combination with a single 20 Gy RT dose greatly reduced tumor growth compared to either treatment alone and resulted in complete tumor rejection in 70% of the mice (42). Besides cGAMP analogues, an interesting discovery are the STING activating properties of PC7A nanovaccine, which consist of E7 peptide or OVA peptide-loaded micelle NPs binding to STING (43). Half of the mice treated with the combination of PC7A and 20 Gy RT were tumor free 60 days after tumor inoculation, compared to none of the mice from the single treatment groups. Treatment efficacy showed to be depended on STING signaling and increased tumor reactive CD8^+^ T cells were observed (43). RT induces cytoplasmic DNA and micronuclei formation which can activate the cGAS/STING pathway. It has been shown that cGAS/STING dependent DNA sensing in DCs is essential in triggering adaptive immunity following RT (42). Other studies, however, report that also cancer cell intrinsic cGAS activation can be important in the induction of an adaptive immune response following RT. cGAMP produced by cancer cells was shown to be transported to DCs *via* gap junctions, resulting in STING activation in these DCs and subsequent type I IFN production (44). One explanation for the beneficial effect of exogenous STING ligand administration on top of RT induced activation could be the numerous regulatory mechanisms that control cGAS-STING pathway activation. TREX1 is a RT inducible dsDNA exonuclease that attenuates the STING signaling cascade (45). More recently, BAF and C9orf72 have been implicated in the regulation of myeloid STING activation (46, 47). It would therefore be interesting to determine if these regulatory mechanisms are upregulated following tumor ablation and whether stimulation of the cGAS/STING pathway upon ablation would be beneficial to achieve an *in situ* cancer vaccine.

However, some degree of caution should be taken as tumor cell intrinsic cGAS/STING activation has been linked to metastases formation (48). This highlights that an appropriate balance and possibly myeloid cell specific STING pathway activation may be required for optimal anti-tumor immunity.

#### Saponin-Based Adjuvants

Antigen cross-presentation by DCs is crucial for CD8^+^ T cell mediated anti-tumor immunity. Although most conventional adjuvants are unable to boost CD8^+^ T cell responses, SBAs are known to be superior in inducing antigen cross-presentation by DCs (1). Cryoablation with co-injection of SBAs, leads to an extremely potent systemic anti-tumor response. These effects are dependent on the ability of SBAs to induce cross-presentation, specifically in CD11b^+^ DCs (49). Additional administration of CpG with SBAs following cryoablation created a highly effective *in situ* cancer vaccine and resulted in the generation of multifunctional T cells able to produce high amounts of pro-inflammatory cytokines (50). The exact mechanism through which SBAs induce cross-presentation remains elusive, although lipid bodies are found to play a crucial role (49). Interestingly, monocyte-derived CD11b^+^ DCs have been implicated to be better in the activation and induction of memory CD8^+^ T cells as compared to cDC1 (51, 52). Therefore, SBAs might be specifically potent in inducing long term immune memory. Besides cryoablation also other ablation therapies, such as RT and HIFU, are of interest for their potential synergy with SBAs.

Altogether, adjuvants are suitable candidates to be applied with tumor ablation to generate an *in situ* cancer vaccine (**Figure 1B1**). More detailed knowledge about effective adjuvant-ablation strategies, such as correct timing and the involved immune subsets, is required to efficiently prime and boost anti-tumor immunity.

### Agonistic Antibodies

DCs can further be activated by cell-cell contact and subsequent signaling *via* members of the immunoglobulin domain-containing receptor family, especially the tumor necrosis factor (TNF) receptor family, such as CD40/CD40 ligand (CD40L) and CD27/CD70. CD40 engagement on DCs by CD40L expressed by CD4^+^ T helper cells or agonistic CD40 mAbs trigger DC activation to provide signals for the licensing and expansion of CD8^+^ cytotoxic T cells (53, 54). CD40 agonistic mAbs have shown synergistic effects in combination with RT. Addition of agonistic anti-CD40 mAb to 10 Gy RT increased survival of mice inoculated with EG7 tumors to 80% as compared to 40% (anti-CD40) and 20% (RT) for the monotherapy regimens (55). All surviving mice treated with the combination therapy were resistant to a subsequent re-challenge, indicative for immune memory. Similar results have been achieved using a Panc02 tumor model where combination therapy not only limited primary tumor growth, but also growth of an untreated contralateral tumor (56). In the latter model, agonistic CD40 therapy worked best when combined with a hypo-fractionated RT regimen (5 Gy single dose). Moreover, timing of anti-CD40 mAb administration relative to RT treatment was crucial for its efficacy as administration prior to RT did not show beneficial effects (57). TLR agonists, such as CpG and Poly-IC, are known to upregulate the expression of CD40 on human pDCs as well as myeloid DCs (58, 59). Poly-IC is especially interesting as in combination with anti-CD40 mAb it induced the highest percentage of OVA-specific T cells relative to other TLR agonists (60). This can possibly be explained by the upregulation of CD70, the ligand for the T cell co-stimulatory receptor CD27, following stimulation with Poly-IC and anti-CD40 mAb (61). It would therefore be interesting to investigate the efficacy of the triple combination of agonistic mAbs plus adjuvants and ablative therapies.

Besides DCs, also T cells express multiple co-stimulatory receptors, including CD27, OX40 and CD137 (4-1BB). Ligation of these receptors delivers co-stimulatory signals necessary for full T cell activation (62–65). RT induces upregulation of OX40 expression on CD4^+^ and CD8^+^ T cells as well as CD137 on CD8^+^ tumor infiltrating T cells and thus works in concert with agonistic OX40 or CD137 therapy (66–68). CD137 expressing CD8^+^ T cells are also highly positive for PD-1, and RT plus agonistic CD137 therapy benefits from additional anti-PD-1 mAbs to block negative feedback by PD-L1 (69). Combining multiple different immune activation strategies which complement each other is an appealing approach to further stimulate the immune response. Noteworthy, combinations of CpG plus RT, agonistic OX40 mAbs plus RT as well as CpG plus agonistic OX40 mAbs have shown synergistic effects in their ability to limit tumor growth, making the combination of CpG, agonistic OX40 with RT or other ablative therapies an interesting approach to explore (29, 70, 71).

To date, all agonistic antibodies investigated have shown promising results in combination with RT and the addition of adjuvants might further improve their function (**Figure 1B2**). Whether these agonistic antibodies also synergize with other ablative modalities remains to be determined.

## Counteracting the Immunosuppressive TME

Tumors have evolved several mechanisms to instigate an immunosuppressive TME, parts of which may admix with the antigen depot upon ablation. Successful *in situ* cancer vaccines may, in addition to immune activation strategies, also require approaches that counteract the immunosuppressive microenvironment. Immune suppression networks consist of immune suppressive cells including Tregs and MDSCs, immunosuppressive cytokines like TGFβ and IL-10, as well as enhancement of co-inhibitory molecules such as CTLA-4 or PD-1 on T cells. Targeting immunosuppressive cells has emerged as important approach to counteract the immunosuppressive TME, which is discussed in detail elsewhere (72–74). In the next sections, we will discuss strategies to counteract the immunosuppressive molecules, with a focus on cytokine and immune checkpoint blockade, applied together with *in situ* tumor ablation.

### Immunosuppressive Cytokines

Immune suppressive cytokines, such as TGFβ and IL-10, are a major obstacle in generating effective anti-tumor immunity. They are often produced by tumor cells and immune suppressive cell subsets, such as Tregs and MDSC (75). RT is known to increase the amount of active TGFβ. TGFβ is initially produced in its latent form containing a pro-domain, dissociation of this domain makes the protein become active. Oxygen radicals produced following RT promote this dissociation resulting in more active TGFβ (76, 77). Thermal ablation at temperatures above 65 °C can lead to denaturation of proteins, potentially including part of immune suppressive cytokines, such as TGFβ or IL-10. Strategies that block inhibitory signaling through antagonistic antibodies, as well as scavenging of inhibitory cytokines themselves are ways to alleviate their inhibitory function (**Figure 1B3**).

Scavenging of TGFβ using antibodies limits growth of treated and untreated tumors following 5 x 6 Gy RT (78). This combination therapy increased DC maturation evidenced by an increase in CD40^+^CD70^+^ DCs. Furthermore, the combination increased the production of IFNγ by dLN-derived CD8^+^ T cells following *ex vivo* tumor antigen stimulation. Lastly, the percentage of PD-1^+^ and PD-L1/2^+^ cells in the tumor increased upon combination therapy, highlighting the induction of additional immune escape mechanisms. Inclusion of anti-PD-1 mAbs indeed further improved tumor control. Other successful TGFβ neutralizing approaches include recombinant TGFβ receptor (TGFβR) fused to an Fc-tail (79). The mechanism behind the anti-tumor effect of TGFβ scavenging is not solely immune mediated as TGFβ has pleiotropic functions, such as in wound healing and DNA repair, which could play a role with the anti-tumor effect (80, 81).

The cytokine IL-10 inhibits macrophage pro-inflammatory cytokine production, limits DC antigen presentation, and dampens T and NK cells effector function (82). Interestingly, some studies, however, report an increase in intra-tumoral cytotoxic CD8^+^ T cells upon IL-10 delivery to the tumor (83). This can be explained by the ability of IL-10 to limit IFN-γ production by DCs, which is crucial for activation induced T cell apoptosis (84). All in all, efficacy of scavenging or blockade of anti-inflammatory factors will probably dependent on the choice of ablative therapy and state of the immune response when applied.

### Immune Checkpoints

To shift the balance of the TME away from immunosuppression, mAbs can be applied to block inhibitory immune checkpoint receptors or their ligands (85). Relieving immunosuppression of adaptive immune cells has been extensively studied, and mAbs targeting CTLA-4 or PD-1/PD-L1 can enhance T cell immunity generated by ablation (**Figure 1B4**). CTLA-4 blockade allows CD80 and CD86 co-stimulatory molecules to be available for CD28, lowering the threshold for T cell activation (86). Anti-CTLA-4 mAbs also cause intra-tumoral Treg depletion or modulation of their suppressive functions (87, 88). CTLA-4 blockade synergized with different forms of thermal tumor ablation, resulting in significant amounts of active tumor-specific T cells and the ability to reject secondary or re-challenged tumors (89–91). Data from a pilot study conducted in breast cancer patients that received cryoablation and anti-CTLA-4 mAb showed good tolerability and promising efficacy (92). In the line of relieving immunosuppression, Treg depletion using anti-CD25 mAb enhances the anti-tumor response after RT, RFA and cryoablation, indicated by the increased presence of IFNy producing T cells after combination therapy in case of the latter two (89, 93, 94). Currently various clinical trials are ongoing testing the potential of *in situ* ablation and checkpoint blockade in different solid malignancies.

PD-L1 is often highly expressed on tumor cells and tumor associated myeloid cells. PD-L1 can be induced by pro-inflammatory cytokines and is frequently upregulated in response to *in situ* tumor ablation (95, 96). Engagement of PD-1^+^ T cells with its ligands leads to suppression of T cell effector mechanisms and mAbs that block the PD-1/PD-L1 axis are aimed at reinvigorating these exhausted T cells. RFA treatment of a localized tumor increased T cell infiltration in a distant tumor in both tumor-bearing mice as well as human patients (97). However, these tumors quickly overcame T cell cytotoxicity by inhibiting infiltrating T cells *via* upregulation of PD-L1 expression. In the murine setting, combining RFA with anti-PD-1 mAbs increased the tumor antigen-specific T cell response, and synergistically inhibited growth of distant tumors (97). Strikingly, incomplete RFA tumor ablation limited the efficacy of anti-PD-1 immunotherapy (98). The authors demonstrated that incomplete ablation induced local inflammation and resulted in accumulation of immunosuppressive myeloid cells in the residual tumor, which inhibited T cell functionality. Targeting the CCL2/CCR2 pathway, responsible for the recruitment of these immunosuppressive myeloid cells, enhanced anti-tumor immunity in the residual tumor, and thereby overcame the resistance to anti-PD-1 therapy.

Synergy between adaptive immune checkpoint blockade and RT has been demonstrated in multiple different preclinical tumor models (95, 99–102). However, RT dose and fractionation regimens as well as the timing of checkpoint blockade administration in conjunction with RT that would result in the most optimal anti-tumor immune response differ and warrant further study (103). Several other promising novel adaptive immune checkpoint molecules are actively being investigated, including TIM-3, LAG-3, TIGIT and VISTA (104), which could also be potential targets.

Recent studies have indicated that tumor cells exploit sialoglycan–Siglec interactions to modulate cytotoxic T cell as well as myeloid cell function, contributing to an immunosuppressive TME (105). Interference with the sialoglycan-Siglec axis by inhibiting the sialic acid synthesis pathway resulted in enhanced anti-tumor immunity and limited tumor outgrowth (106). Next to Siglec receptors, studies have highlighted innate immune checkpoints as interesting therapeutic targets. One of these checkpoints is the signal-regulatory protein α (SIRPα)-CD47 axis. CD47 is often overexpressed on tumor cells and interacts with SIRPα on myeloid cells to trigger a ‘don’t eat me’ signal (107). Blocking SIRPα-CD47 interactions alleviates inhibitory signaling resulting in improved tumor cell clearance. Besides, murine models suggest that adaptive immunity contributes to tumor control upon targeting the SIRPα-CD47 pathway (108–110). This can be a direct effect of the SIRPα-CD47 pathway on T cell function or an indirect mechanism by which SIRPα-CD47 pathway blockade affects the capacity of myeloid cells to activate T cells. Interestingly, efficacy of CD47 blockade was shown to largely depend on DNA sensing, specifically in DCs (41). Interference with sialoglycan–Siglec interactions as well as innate immune checkpoints should be further explored in the context of *in situ* tumor destruction.

## Future Perspective: Multifunctional Antibody Development and *In Situ* Tumor Ablation

**Figure 2:**
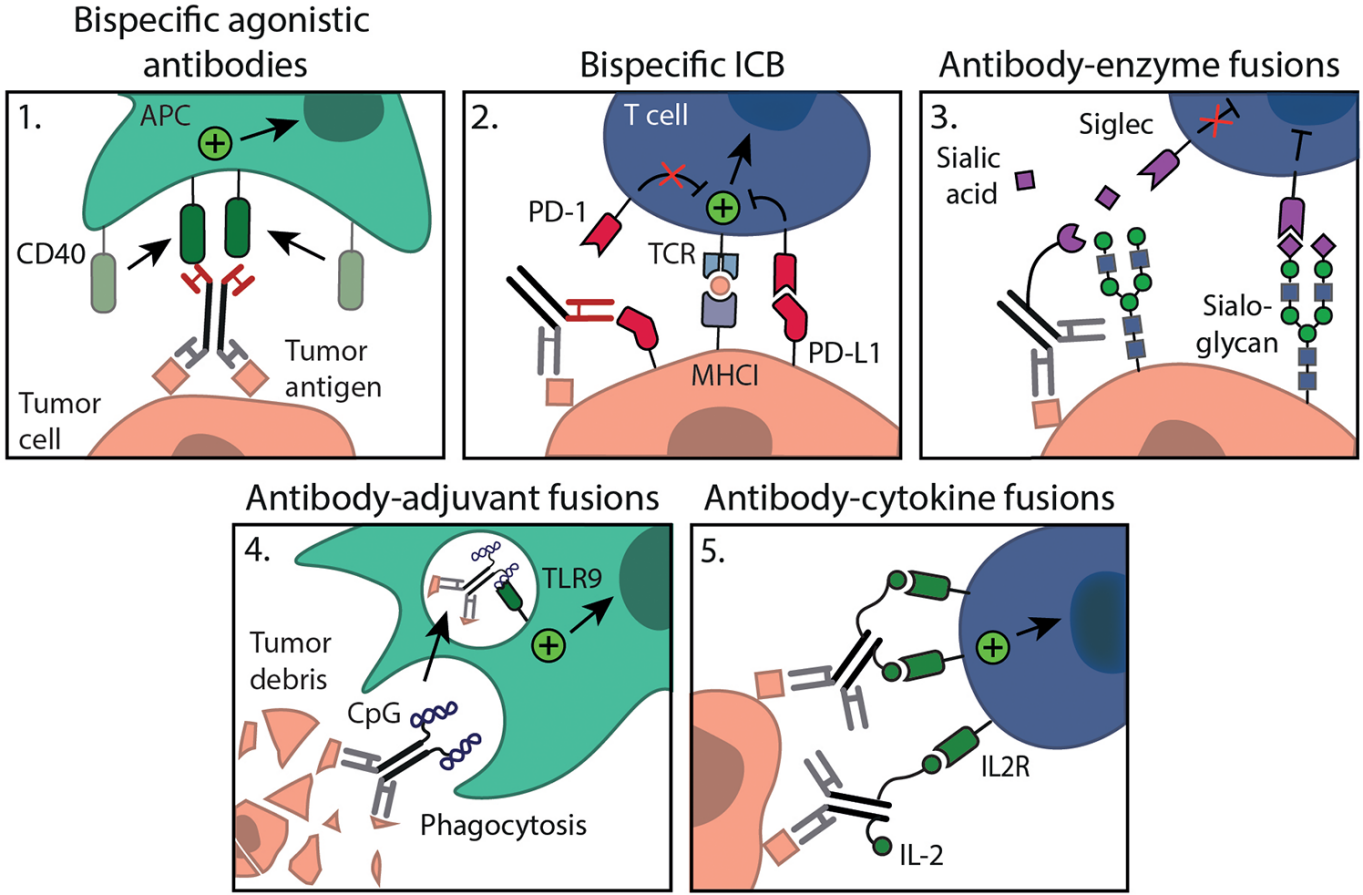
Multifunctional antibody formats for combination with *in situ* tumor ablation. Administration of 1. bispecific agonistic antibodies (e.g. anti-MSLN-CD40) or 4. antibody-adjuvant fusions (e.g. chTNT3-CpG) will lead to local APC activation. Interventions such as 2. bispecific ICB (e.g. PD-L1xErbB2 antibody) may further stimulate myeloid as well as T cell immunity specifically within the TME; 3. antibody-enzyme fusions allow tumor specific sialoglycan degradation (e.g. anti-HER2 mAb-sialidase); 5. antibody-cytokine fusions (e.g. anti-GD2-IL2) will result in targeted cytokine delivery ensuring local immune cell activation, all are aimed at relieving local immunosuppression.

The tumor exists in a dynamic microenvironment that co-influences anti-tumor immune responses. Strategies that simultaneously modulate multiple key processes in the anti-tumor immune response will likely work synergistically. Recent advances in antibody engineering have resulted in new antibody formats that can exert distinct effector functions (111). Besides, multifunctional antibodies can be used to direct immunomodulators specifically to the relevant locations, limiting systemic exposure and increasing tumor specificity. Multifunctional antibodies come in various molecular varieties, ranging from linked Fab fragments to full antibodies with an Fc-tail to preserve native antibody functions, such as antibody-dependent cellular cytotoxicity (ADCC)/phagocytosis, complement-mediated lysis and improved circulation half-life (111). Multifunctionality can be achieved by combining different antibody variable domains, recognizing different epitopes, e.g. bispecific antibodies. Alternatively, receptors or immunomodulatory molecules can be attached to antibodies *via* protein-linkers, acquiring multiple specificity in a different manner. Most of these multifunctional antibody formats are in (pre)clinical development and not yet applied in context with tumor ablation. We will here review antibody formats that could be beneficial in combination with tumor ablation to create an *in situ* cancer vaccine (**Figure 2**).

Bispecific antibodies come in various flavors and can target different antigens either on the same cell or on two different cell types. Most known bispecific antibodies in preclinical and clinical development are engaging T cells, binding CD3 and a relevant tumor antigen, to induce tumor cell killing (111). Alternatively, bispecific antibodies harboring an agonistic arm and a tumor targeting arm are developed (112, 113). These bispecific agonistic antibodies ensure tumor localization and allow cross-linking without the need for Fc-receptors to exert its agonistic function (**Figure 2.1**). Instead they rely on a tumor antigen for cross-linking, making activation fully tumor cell dependent. For example, the bispecific antibody LB-1, which is specific for the tumor antigen MSLN and mouse CD40 showed preferential DC activation *in vitro* only when cultured with MSLN expressing tumor cells. *In vivo* application limited tumor growth of an MSLN expressing tumor to a similar extent as a conventional agonistic anti-CD40 mAb. The bifunctional molecule, however, showed less systemic activation and toxicity as compared to anti-CD40 therapy (112, 113). In addition to these there are also bispecific agonistic antibody constructs targeting two co-stimulatory receptors at once, CD137 and OX40, or a co-stimulatory receptor (OX40) and immune checkpoint (CTLA-4) (114, 115).

The success of immune checkpoint mAbs prompted the development of bispecific immune checkpoint formats, such as the PD-L1xErbB2 antibody (**Figure 2.2**). This bispecific antibody reduced tumor growth and increased tumor rejection rate compared to the combination of anti-PD-L1 and anti-ErbB2 mAb therapy, which was dependent on CD8^+^ T cells and IFNγ (116). The bispecific antibody was constructed with a mIgG2a Fc backbone and the authors describe that ADCC and complement action could be potential mechanisms (116). Alternatively, bispecific antibodies binding two distinct immune checkpoints, such as PD-1/PD-L1, CTLA-4, LAG-3 or TIM-3 are also interesting options to explore (117, 118). Besides, innate immune checkpoints are explored in bispecific antibody formats. Bispecific mAbs consisting of a low-affinity anti-CD47 arm combined with a high-affinity tumor antigen arm ensure that blockade of CD47 only occurs on tumor cells, which co-express both antigens, resulting in improved phagocytosis of target cells and leaving healthy CD47 expressing cells unharmed (119). Bispecific antibodies show potent anti-tumor activity and warrant further study in combination with ablation. As Siglec receptors are regarded as novel immune checkpoints, it would be interesting to explore Siglec targeting antibodies in bi- or multispecific formats.

Alternatively, multispecificity can be achieved through the linking of recombinant receptors/ligands or immunomodulatory molecules to antibodies. To this end, endogenous SIRPα domains are engrafted to a tumor antigen specific antibody (120, 121). Binding of the antibody to tumor antigen specific cells allows binding of the SIRPα domain to CD47 on these same cells. Thereby, the interaction of CD47 with endogenous SIRPα expressed on myeloid cells is prevented, restoring the phagocytic capacity of myeloid cells (120, 121). In the context sialoglycan–Siglec axis, a recently developed multifunctional antibody consisting of a sialic acid-cleaving enzyme (sialidase) fused to an anti-HER2 antibody, aims to degrade sialoglycans in a tumor-specific manner (**Figure 2.3**) (122). In a syngeneic orthotopic HER2^+^ breast cancer model, anti-HER2 antibody-sialidase conjugates delayed tumor growth and enhanced immune infiltration, leading to prolonged survival of mice. Using the HER2^+^ B16D5 melanoma tumor model and Siglec-E^-/-^ mice, the authors showed that the effect was dependent on functional Siglec-E, a receptor highly expressed on tumor-infiltrating myeloid cells (122). These studies indicate that multifunctional antibodies aimed at reversing the immunosuppressive TME are potentially effective. Also, immune activation strategies, such as adjuvants can be incorporated into mAb conjugates (**Figure 2.4**). One such antibody is chTNT3-CpG, which is specific for extracellular DNA/histones (123), often present following ablative therapy. Systemic intraperitoneal (i.p.) administration of chTNT3-CpG resulted in delayed tumor development in both the Colon 26 adenocarcinoma and B16 tumor model, whereas i.p. administration of the chTNT3 antibody or CpG alone failed to show efficacy, again showing the added value of tumor targeting capacities of multifunctional mAbs (123).

Trafficking of APCs to the ablation site where they can capture and process antigens for (cross-) presentation is of importance for an *in situ* cancer vaccine. To this end, antibodies conjugated with DC growth factors, such as GM-CSF or FLT3L, are of interest to expand and redirect DC subsets to the ablation site (124, 125). In fact, preclinical data showed that FLT3L in combination with RT in a mammary carcinoma model can help boost the abscopal effect (126). GM-CSF has been coupled to anti-HER2/neu and demonstrated anti-tumor activity in a HER2/neu expressing colon adenocarcinoma model (127). Several other cytokines including interleukin 2 (IL-2), IL-12 and type I IFN have been fused to antibodies (**Figure 2.5**). The use of antibody-cytokine fusions has to potential to concentrate the cytokines at the tumor site, reducing side effects that are observed with systemic pro-inflammatory cytokine administration. IL-2, an important cytokine in the regulation of adaptive T cell responses, has been fused to diverse antibodies targeting relevant tumor proteins, such as hu14.18-IL2 targeting disialoganglioside GD2, huKS-IL2 targeting EpCAM, L19-IL2 targeting fibronectin and NHS-IL2 targeting histone/DNA complexes. IL-2 fusion antibodies were shown to improve responses to *in situ* tumor ablation, resulting in marked tumor reduction (128–130) and curative abscopal effects (131), mediated by CD8^+^ T cells.

Preclinical research demonstrated that combination of 12 Gy RT together with five i.t. injections of hu14.18-IL2 on days 6 to 10 after RT eradicates constitutively GD2 expressing B78 melanoma tumors (132). In the ~70% of mice that were rendered disease-free upon combination therapy, 90% rejected a re-challenge with GD2^high^ B78 melanoma cells. This response of RT and hu14.18-IL2 in melanoma could be augmented by addition of anti-CTLA-4 mAb (133). A recent study further pursued the combination of hu14.18-IL2 and RT as *in situ* cancer vaccination strategy. Voeller et al. demonstrated that neither RT plus hu14.18-IL2 therapy nor the addition of anti-CTLA-4 mAb to the combined therapy regimen caused significant growth inhibition in a GD2^high^ non-immunogenic 9464D neuroblastoma model (134). These observations suggest that the antibody-cytokine mediated therapeutic effect is tumor type dependent. Addition of the adjuvant CpG and anti-CD40 co-stimulatory agonist to the RT, ½ dose hu14.18-IL2 (due to concern for significant toxicities) and anti-CTLA-4 mAb, improved tumor control and 80% achieved complete tumor regression. A clinical phase II study recently demonstrated that hu14.18-IL2 given in combination with GM-CSF and the differentiation inducing agent isotretinoin is safe and tolerable, and showed anti-tumor activity in patients with relapsed/refractory neuroblastoma (135). Several other IL2-antibody fusions have advanced to clinical trials, including huKS-IL2 (136), NHS-IL2 (128) and L19-IL2 (137). The combination of RT (5 x 4 Gy) followed by NHS-IL2 after first-line chemotherapy in metastatic non-small cell lung cancer (NSCLC) patients was well tolerated (128). A phase II trial will investigate the combination of SABRT and L19-IL2 therapy in metastatic NSCLC patients (137).

IL-12, an important CD8^+^ T cell and NK cell cytokine, has been fused to the anti-NHS antibody recognizing histone/DNA complexes. Enhanced tumor uptake of radiolabeled NHS-hIL12 was observed upon RT ablation in rhabdomyosarcoma xenografts (138). Fallon et al. showed that 0.36 Gy RT combined with subcutaneous NHS conjugated with murine IL-12 resulted in superior tumor growth inhibition compared to either treatment alone in a murine LLC lung and MC38 colorectal cancer model (139). Studies combining other ablation types with NHS antibody cytokine fusions are not reported, however would be worthwhile to explore.

Besides interleukins, other pro-inflammatory cytokines, such as type I IFNs, have been coupled to various antibodies. IFNβ fused to anti-EGFR mAb limited growth of mouse EGFR-expressing B16 tumors which were unresponsive to anti-EGFR mAb therapy (140). Furthermore, multifunctional antibodies that simultaneously aim at activating the immune system and counteracting the immunosuppressive TME are promising for future cancer vaccine developments. To this end, anti-PD-L1 was armed with IFNα to simultaneously target both PD-L1 and the IFN-receptor. In different models, anti-PD-L1-IFNα could control advanced tumors as opposed to IFNα-Fc or anti-PD-L1 monotherapy (141). In addition, multifunctional antibodies aimed at blockade of different immunosuppressive pathways are developed, such as the fusion protein M7824, comprising the extracellular domain of human TGFβRII (TGFβ scavenging/trap) linked to the human anti-PD-L1 heavy chain. Combination therapy with M7824 (intravenous, day 2) and RT (3.6 Gy per day, days 0-3) reduced primary as well as untreated secondary tumor growth relative to either treatment alone, indicating the induction of an abscopal effect (142). A phase I trial of M7824 showed a manageable safety profile in patients with heavily pretreated advanced solid tumors and encouraging treatment efficacy (143). Overall, multifunctional antibodies can be created by linking different immunomodulatory molecules with tumor or immune targeting mAbs. Proof-of-concept preclinical studies suggest therapeutic potential of different multifunctional antibody formats and clinical trials showed tolerability and safety. We anticipate that these multifunctional antibodies can work in concert with *in situ* tumor ablation and highlight them as a promising therapeutic strategy to explore.

## Conclusion

*In situ* tumor ablation techniques allow for (neo)antigen loading of DCs without prior knowledge of tumor antigens or epitopes as in conventional DC vaccination. The induction of an efficient immune responses following ablation, however, requires addition of immune stimuli to eradicate local tumors and metastases and to provide long-term protection. Numerous immune activating strategies have shown to be suitable to act in concert with ablation generated tumor debris to achieve *in situ* cancer vaccination. More detailed knowledge about how effective immune activation strategies can work in concert with tumor ablation, such as timing and dose, is required to guide rationale ablation combination strategies. Although *in situ* ablation plus immune activating strategies ensure that the immune system is well instructed and initiated, the immunosuppressive environment that immune cells encounter upon arrival at the TME is still a potential bottleneck. Therefore, additional removal of inhibitory influences provides the possibility to further boost anti-tumor immune responses and enhance *in situ* ablation efficacy.

Multifunctional antibodies stimulating immune activation as well as counteracting immunosuppression can simultaneously affect multiple key processes in the anti-tumor immune response. They hold great promise for targeted cancer treatment with limited systemic toxicities and deserve further exploration as potential strategy to achieve a successful *in situ* cancer vaccine.

## Author Contributions

RB, FS and GA wrote the manuscript. JF, MV and LC reviewed the manuscript. All authors contributed to the article and approved the submitted version.

## Funding

This work was supported by the foundation ‘Villa Joep’, a Radboud Institute for Molecular Life Sciences (RIMLS) PhD grant awarded to GA and a grant from the Dutch Cancer Foundation (KWF) awarded to GA (KUN2015-7604).

## Conflict of Interest

The authors declare that the research was conducted in the absence of any commercial or financial relationships that could be construed as a potential conflict of interest.
